# DICOM-Based Quantification of MRI Artifact from Auditory Implants: a Technical Note Comparing Bone-Conduction- and Cochlear Implants

**DOI:** 10.1007/s00062-026-01675-z

**Published:** 2026-06-02

**Authors:** Benjamin Straub, Susan Arndt, Horst Urbach, Lucas I. Becker

**Affiliations:** 1 Department of Otorhinolaryngology, Head and Neck Surgery, University Medical Center Freiburg, Freiburg, Germany; 2 Department of Neuroradiology, University Medical Center Freiburg, Freiburg, Germany

**Keywords:** Magnetic resonance imaging, Artifact quantification, Bone-conduction implant, Cochlear implant, DICOM

## Abstract

**Purpose:**

MRI in patients with auditory implants is limited by implant-related signal void, geometric distortion, and intravoxel dephasing. Artifact extent is often described qualitatively. We aimed to quantify MRI artifact burden from a novel transcutaneous bone-conduction implant (SentioTM) and to compare it with cochlear implant controls under routine, label-compliant 1.5 T clinical conditions.

**Methods:**

We retrospectively analyzed MRI examinations of patients with a SentioTM implant (*n* = 7) and cochlear implant controls (*n* = 5). All scans were performed at 1.5 T, the manufacturer-approved field strength for the SentioTM Ti implant, on the same scanner using routine transverse turbo spin-echo sequences (T2-weighted and T1-weighted; 5‑mm slice thickness). Artifacts were quantified slice-by-slice with a custom Python-based DICOM pipeline. The artifact-to-brain ratio (ABR, %) and maximum void area (cm^2^) were computed; ABRmax (peak slice) served as patient-level summary. Groups were compared using the Mann-Whitney U test and Cliff’s delta.

**Results:**

SentioTM implants showed smaller and more confined artifacts. Median ABRmax was 1.1% [IQR 0.2] with a median maximum void area of 1.7 cm^2^ [IQR 0.4]. Cochlear implant controls showed larger artifacts (ABRmax 2.6% [IQR 1.0]; median maximum void area 3.8 cm^2^ [IQR 2.0]; *p* = 0.007–0.01; δ = 0.81–0.89). Supratentorial diagnostic image quality outside the immediate implant region was preserved in all T2w/T1w SentioTM examinations and partially preserved in 3/5 cochlear implant examinations.

**Conclusion:**

Quantitative artifact assessment using ABRmax is feasible in routine 1.5 T MRI. Under identical protocol conditions, the SentioTM implant produced a smaller and more localized MRI artifact burden than the cochlear implant controls examined here. Routine turbo spin-echo imaging remained diagnostically useful outside the implant region.

**Supplementary Information:**

The online version of this article (https://doi.org/10.1007/s00062-026-01675-z) contains supplementary material, which is available to authorized users.

## Introduction

Auditory implants contain magnets and metallic components that generate local field inhomogeneity and cause implant-related MRI artifacts, including signal void, intravoxel dephasing, and geometric distortion [1–3]. Artifact extent depends on implant design, MRI sequence, and head position, and typically increases with field strength [4–6]. Direct quantitative comparisons across implant types using identical scanners and protocols are scarce. Bone-conduction and cochlear implants are used in different clinical indications and are not clinically interchangeable; the present comparison is therefore intended as a protocol-matched technical reference for implant-related MRI artifact burden, not as a comparison of therapeutic indications. We introduce a simple DICOM-based, slice-wise artifact metric, the artifact-to-brain ratio (ABR, %), and apply it to a protocol-matched in vivo comparison of a novel transcutaneous bone-conduction implant (SentioTM Ti) and cochlear implant controls by two different manufacturers (see Supplementary Material) at 1.5 T.

## Materials and Methods

This retrospective study included patients with a SentioTM Ti (Oticon Medical AB, Askim, Sweden) transcutaneous bone-conduction implant (*n* = 7) and cochlear implant controls (*n* = 5) examined at our institution. The cochlear implant control group comprised four different implant models: M12xx Series Flex 26 (MED-EL, *n* = 2), Nucleus Contour (Cochlear, *n* = 1), CI622 (Cochlear, *n* = 1), and CI612 (Cochlear, *n* = 1). Implant positioning and absence of perioperative complications were confirmed from surgical reports. No implant-related adverse events during MRI were documented (see Supplementary Material).

All MRI examinations were performed on the same 1.5 T system (Siemens Avanto, Siemens Healthineers, Erlangen, Germany; maximum gradient amplitude 45 mT/m, maximum slew rate 200 T/m/s) using a standard 8‑channel head coil according to manufacturer labeling. The SentioTM Ti implant is MR Conditional at 1.5 T; therefore, the study was restricted to the currently approved field strength for this device. Axial T1-weighted turbo spin-echo sequences were acquired in all cases. Axial T2-weighted turbo spin-echo sequences were acquired in 6/7 Sentio cases; one Sentio examination with 3D FLAIR was reported descriptively in the Supplementary Material.

Inclusion required a postoperative interval of at least 6 weeks. The external speech processor was removed before MRI. The axial T1- and T2-weighted TSE sequences used 5‑mm slice thickness with a 0.5-mm interslice gap; the single 3D FLAIR sequence used 3‑mm slices. Further sequence parameters are provided in the Supplementary Material.

Artifact quantification was performed using a standardized DICOM-based pipeline implemented with custom Python 3 scripts using standard DICOM and image-processing libraries. Pixel spacing was extracted from DICOM metadata to convert pixel counts to absolute areas. For each axial slice s, an intracranial envelope mask was derived by intensity-based foreground detection and morphological refinement; this mask defined the normalization area Aenv(s). The implant-related artifact was operationally defined as near-complete signal void within the intracranial envelope. Void pixels were segmented using a uniform near-noise intensity threshold, followed by removal of small isolated components not attributable to the dominant implant-related artifact. The boundary between artifact and usable signal was therefore defined by the same threshold-based algorithm in all cases rather than by manual delineation. ABR was calculated as ABR(s) = 100 × Avoid(s)/Aenv(s), where Avoid(s) is the void area on slice s. ABRmax was defined as the maximum ABR(s) across slices and used as a patient-level summary metric. The algorithm was applied identically to all datasets without manual re-segmentation; visual review by two board-certified neuroradiologists served as a quality control step.

Group comparisons were performed using the Mann-Whitney U test. Effect size was expressed as Cliff’s delta. Data are reported as median [IQR].

## Results

**Fig. 1:**
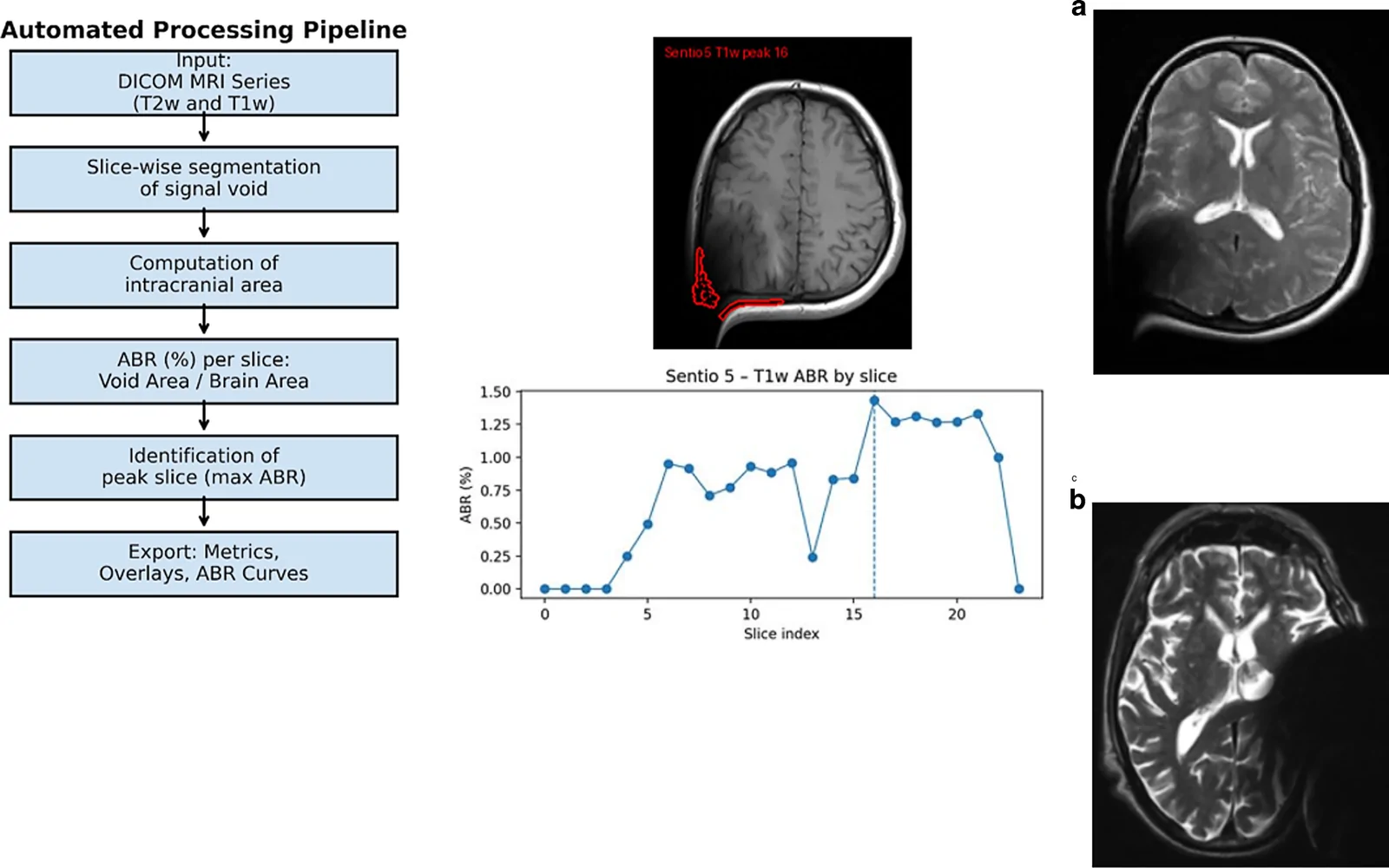
DICOM-based artifact quantification and representative comparison. Workflow of slice-wise intracranial masking, void segmentation, and ABRmax determination. **a** SentioTM implant (T2w) with confined implant-related artifact. **b** Cochlear implant (T2w) with more extended artifact at comparable level

SentioTM implants demonstrated smaller and more sharply confined artifacts on standard TSE sequences, typically limited to one to two slices at the implant level. Cochlear implants showed larger artifacts extending over three to five slices. With the 5‑mm TSE slice thickness and 0.5-mm interslice gap, this corresponded approximately to 5–11 mm craniocaudal coverage for SentioTM implants and 16–27 mm for cochlear implant controls. Quantitative metrics are summarized in Table 1; an illustrative image is Fig. 1. Inter-group difference was significant (ABRmax *p* = 0.007; δ = 0.83). One Sentio examination acquired with 3D FLAIR showed the highest ABRmax and void area within the Sentio cohort; exclusion of this case did not change the direction of the group difference.

**Table 1:** Quantitative artifact metrics (median [IQR])

Metric	SentioTM	Cochlear implant	*p*-value	Cliff’s δ
*ABRmax (%)*				
T2-weighted TSE	1.1 (0.2)	2.6 (1)	0.007	0.83
T1-weighted TSE	1.0 (0.2)	2.4 (1)	0.009	0.81
*Void area (cm*^*2*^*)*				
T2-weighted TSE	1.7 (0.4)	3.8 (1.)	0.01	0.86
T1-weighted TSE	1.5 (0.4)	3.4 (2)	0.007	0.89
*Slices with prominent artifact*	1–2	3–5	–	–
*Supratentorial interpretability*	Preserved (all)	Partially preserved (3/5)	–	–
*Infratentorial interpretability*	Not preserved (all)	Not preserved (all)	–	–

## Discussion

Our protocol-matched 1.5 T data show a lower artifact burden for the SentioTM bone-conduction implant compared with cochlear implant controls. This comparison should be interpreted as a technical reference rather than a comparison of clinical indications, because bone-conduction and cochlear implants serve different patient populations and are not therapeutic alternatives in many cases. However, a partial overlap in indications may exist when bone-conduction implants are used as contralateral routing of signal (CROS) systems. The observed difference is technically plausible because magnet concept, implant configuration, and head position are major determinants of artifact geometry [1–3]. However, our study was not designed for component-level attribution, and published evidence on transcutaneous bone-conduction systems remains limited and is often derived from cadaver experiments or studies using dedicated artifact-reduction techniques [8–10]. Therefore, generalization beyond the specific devices and protocol examined here requires caution.

In future clinical work, the ABRmax tool may be useful primarily as a standardized documentation and comparison tool rather than as a stand-alone pre-MRI prediction model. Quantifying artifact burden on routine DICOM data may support communication of image limitations, selection of follow-up MRI protocols, comparison of implant designs or artifact-reduction strategies, and longitudinal assessment when repeated MRI examinations are required. With larger reference datasets, similar quantitative metrics may also help estimate expected artifact extent before MRI for specific implant-protocol combinations.

ABRmax provides a pragmatic, protocol-matched descriptor derived from standard DICOM images. Compared with simple artifact diameter, ABRmax normalizes void area to the intracranial envelope on the same slice, which reduces dependence on head size and slice level and directly reflects the proportion of the slice rendered non-diagnostic by complete signal void. In contrast to volumetric artifact analysis, it can be obtained from routine two-dimensional TSE acquisitions without requiring isotropic data or additional acquisition. ABRmax should therefore be viewed as a complementary standardized metric rather than a replacement for regional diagnostic assessment or geometric-distortion analysis [1–3]. The automated threshold-based segmentation reduces operator dependency; visual review served as quality control and did not introduce manual corrections. The single 3D FLAIR case illustrates that intensity-based void segmentation is sequence dependent and reinforces the need for sequence-matched quantitative comparisons [1, 2]. Metal-artifact-reduction strategies (e.g., O‑MAR, SEMAC-based approaches) can further reduce artifact size and improve skull-base assessment, but were not available in our routine protocol [7–9].

Limitations include the small retrospective cohort, single scanner, single field strength, and absence of dedicated artifact-reduction sequences. The single-field-strength design reflects current manufacturer labeling for the SentioTM Ti implant, which is MR Conditional at 1.5 T, and should therefore not be interpreted as lack of evaluation of an approved 3 T use case. Generalization to other implant models, scanner platforms, field strengths, or metal-artifact-reduction protocols requires caution. The comparison of bone-conduction and cochlear implants is technical and protocol-matched, but the devices are clinically indicated for different patient populations. ABRmax captures signal void on the peak slice and does not quantify partial signal loss, distortion of anatomy without complete void, or three-dimensional artifact volume. Formal lesion-detection performance and formal inter-reader reliability were not tested.

## Conclusion

Under routine, label-compliant 1.5 T imaging, the SentioTM implant produced a smaller and more localized MRI artifact burden than the cochlear implant controls examined here. This protocol-matched technical comparison should not be interpreted as comparing clinical indications between device classes. ABRmax is a feasible and reproducible metric for standardized artifact quantification in routine neuroradiologic MRI and may support documentation and comparison of artifact burden across implant-protocol combinations.

## Supplementary Information


ESM DICOM-Based Quantification of MRI Artifact from Auditory Implants.


## Data Availability

The DICOM-based pipeline is available from the corresponding author on reasonable request. Raw imaging data cannot be shared publicly due to patient privacy regulations.
