# Supplementary material for: DICOM-Based Quantification of MRI Artifact from Auditory Implants: a Technical Note Comparing Bone-Conduction- and Cochlear Implants
Source: Clin Neuroradiol. 2026 Jun 2;36(3):1113–7. doi: 10.1007/s00062-026-01675-z (PMC13522037; doi:10.1007/s00062-026-01675-z)
Supplement: Supplementary file 1 [file 62_2026_1675_MOESM1_ESM.docx]

**Electronic Supplementary Material**

**DICOM-Based Quantification of MRI Artifact from Auditory Implants**

**S1. MRI Sequence Parameters**

All MRI examinations were performed on a 1.5 T scanner (Siemens Avanto) using a standard head coil. The detailed sequence parameters are listed below. Values were extracted from DICOM metadata; in-plane FOV computed as Columns × PixelSpacing and rounded to nearest mm.

**Table S1. MRI sequence parameters**

**S2. Patient-Level Quantitative Data**

All artifact metrics were calculated using the standardized DICOM-based pipeline described in the main manuscript.

**Table S2. Patient-level artifact metricsT2-weighted TSE / 3D FLAIR**

| **ID** | **Implant** | **Sequence** | | **ABR_max_ (%)** | **Void (cm²)** | **Artifact-positive slices** |
| --- | --- | --- | --- | --- | --- | --- |
| S1 | Sentio™ Ti, Oticon Medical AB | T2 TSE | | 0.9 | 1.4 | 1 |
| S2 | Sentio™ Ti, Oticon Medical AB | T2 TSE | | 1.0 | 1.5 | 1 |
| S3 | Sentio™ Ti, Oticon Medical AB | T2 TSE | | 1.1 | 1.7 | 2 |
| S4* | Sentio™ Ti, Oticon Medical AB | 3D FLAIR | | 3.7 | 7.3 | 5 |
| S5 | Sentio™ Ti, Oticon Medical AB | T2 TSE | | 1.1 | 1.7 | 2 |
| S6 | Sentio™ Ti, Oticon Medical AB | T2 TSE | | 1.2 | 1.9 | 1 |
| S7 | Sentio™ Ti, Oticon Medical AB | T2 TSE | | 1.3 | 2.1 | 2 |
| C1 | M12xx Series Flex 26, MED-EL | T2 TSE | | 1.9 | 3.0 | 3 |
| C2 | Nucleus Contour, Cochlear | T2 TSE | | 2.2 | 3.2 | 3 |
| C3 | CI622, Cochlear | T2 TSE | | 2.6 | 3.8 | 4 |
| C4 | CI612, Cochlear | T2 TSE | | 3.0 | 4.5 | 5 |
| C5 | M12xx Series Flex 26, MED-EL | T2 TSE | | 3.1 | 5.0 | 4 |
| T1 weighted TSE | | | | | |  |
| **ID** | **Implant** | **ABR (%)** | **Void (cm²)** | | **Artifact-positive slices** |  |
| S1 | Sentio™ Ti, Oticon Medical AB | 0.8 | 1.2 | | 1 |  |
| S2 | Sentio™ Ti, Oticon Medical AB | 0.9 | 1.3 | | 1 |  |
| S3 | Sentio™ Ti, Oticon Medical AB | 1.0 | 1.5 | | 1 |  |
| S4 | Sentio™ Ti, Oticon Medical AB | 1.1 | 1.7 | | 2 |  |
| S5 | Sentio™ Ti, Oticon Medical AB | 1.0 | 1.5 | | 2 |  |
| S6 | Sentio™ Ti, Oticon Medical AB | 1.1 | 1.7 | | 1 |  |
| S7 | Sentio™ Ti, Oticon Medical AB | 1.2 | 1.9 | | 2 |  |
| C1 | M12xx Series Flex 26, MED-EL | 1.8 | 2.7 | | 3 |  |
| C2 | Nucleus Contour, Cochlear | 2.0 | 2.9 | | 3 |  |
| C3 | CI622, Cochlear | 2.4 | 3.4 | | 4 |  |
| C4 | CI612, Cochlear | 2.8 | 4.1 | | 5 |  |
| C5 | M12xx Series Flex 26, MED-EL | 2.9 | 4.6 | | 4 |  |

Patient identifiers are anonymized.
FLAIR case (S4) marked clearly by asterisk (*).

**S3. Patient characteristics and MRI-related safety assessment**

**Table S3. Patient characteristics and MRI-related safety assessment**

| **Parameter** | **Sentio^TM^ (n = 7)** | **Cochlear implant (n = 5)** |
| --- | --- | --- |
| Male : female | 29% : 71% | 40% : 60% |
| Operative center | Single tertiary referral center | Single tertiary referral center |
| Implant system | Sentio active bone-conduction implant | Standard CI |
| Revision surgery | 0 | 0 |
| Postoperative interval at MRI | 7–16 months | 6–18 months* |
| External processor removed during MRI | Yes (all) | Yes (all) |
| Compression bandage during MRI | No | No |
| MRI-related pain/discomfort | 0 | 0 |
| Implant-related adverse events during MRI | 0 | 0 |

*****Range.


**S4. Additional Illustrative Images**

Fig. S1. Sequence-dependent artifact metrics in bone-conduction and cochlear implants. Artifact-to-brain ratio (ABR, %) and maximum void area (cm²) in Sentio and cochlear implant cohorts. Upper panels show T2-weighted TSE data (with one 3D FLAIR case indicated separately and excluded from primary protocol-matched T2w/T1w-TSE analysis); lower panels show T1-weighted TSE data. Individual values are displayed with jittered points; diamonds represent medians and group sizes are indicated in each panel.

**S5. Data Availability**

The DICOM-based pipeline is available upon reasonable request. Due to patient privacy regulations, raw imaging data cannot be shared publicly.
